# Superfœtation, or Twin Conception?

**Published:** 1878-06

**Authors:** T. A. Scott

**Affiliations:** Warren Co., Ill.


*Superfœtation or Twin Conception?*

I was called, March 19th, to attend Mrs. D., aged 41 years, in her eleventh confinement. In due time she was delivered of a fine, healthy female child, weighing ten pounds. After I had tied the cord and handed the child to the nurse, I proceeded to ascertain if the placenta had been expelled, and, on introducing my finger into the vagina, I found there another fœtus. On examination, I discovered it to be about four months old, and in a perfectly healthy condition, there being no sign whatever of decomposition, but, on the contrary, all the appearance of having been alive when labor set in.

Its membranes were entire when expelled, but I neglected to make any examination as to the insertion of its umbilical cord, and cannot say whether there was more than one placenta. There was nothing else about the case in any way unusual, and the mother made a good recovery, the child also doing remarkably well.

Query. Was this originally a twin conception, or was it a case of superfœtation?

T. A. Scott.

Warren Co., Ill.

---

External use of Tincture of Belladonna in Night-sweating.—Dr. J. T. Nairne, (*British Medical Journal.*)

For some little time past I have employed the common pharmacopœial tincture of belladonna for sponging the body in cases of phthisical and excessive sweating, and invariably with marked benefit. So far as my experience goes, I have found it very much better than anything else; if applied before a sweating comes on, it prevents it; if during the sweating, it almost immediately controls it. Two teaspoonfuls of the tincture mixed with an equal quantity of whisky are quite sufficient (applied with the hand) to cover the whole body and produce the desired effect. I have adopted this method of treatment in my last cases of scarlet fever, which have all done well; but they have not been numerous enough to justify any definite opinion of the value of belladonna applied in this manner.

Abuses of Medical Charities.—At the last meeting of the Medical Society of the county of New York, Dr. Geo. Witherell read a report, prepared by the Committee on the Abuses of Medical Charities. In this, it was stated that in Philadelphia, during 1876, one out of every five inhabitants was treated gratuitously at the dispensaries or hospitals; in Boston, during the same period, one out of every four, and in New York also one out of every four. It was shown that only 12 out of 152 applicants for free treatment, whose cases were inquired into, were proper objects of charity.—*Hospital Gazette*, May 2, 1878.

